# Dissociable Genetic Contributions to Error Processing: A Multimodal Neuroimaging Study

**DOI:** 10.1371/journal.pone.0101784

**Published:** 2014-07-10

**Authors:** Yigal Agam, Mark Vangel, Joshua L. Roffman, Patience J. Gallagher, Jonathan Chaponis, Stephen Haddad, Donald C. Goff, Jennifer L. Greenberg, Sabine Wilhelm, Jordan W. Smoller, Dara S. Manoach

**Affiliations:** 1 Department of Psychiatry, Massachusetts General Hospital, Harvard Medical School, Boston, Massachusetts, United States of America; 2 Athinoula A. Martinos Center for Biomedical Imaging, Harvard Medical School, Charlestown, Massachusetts, United States of America; 3 Center for Human Genetics Research, Massachusetts General Hospital, Harvard Medical School, Boston, Massachusetts, United States of America; University of Texas at Dallas, United States of America

## Abstract

**Background:**

Neuroimaging studies reliably identify two markers of error commission: the error-related negativity (ERN), an event-related potential, and functional MRI activation of the dorsal anterior cingulate cortex (dACC). While theorized to reflect the same neural process, recent evidence suggests that the ERN arises from the posterior cingulate cortex not the dACC. Here, we tested the hypothesis that these two error markers also have different genetic mediation.

**Methods:**

We measured both error markers in a sample of 92 comprised of healthy individuals and those with diagnoses of schizophrenia, obsessive-compulsive disorder or autism spectrum disorder. Participants performed the same task during functional MRI and simultaneously acquired magnetoencephalography and electroencephalography. We examined the mediation of the error markers by two single nucleotide polymorphisms: dopamine D4 receptor (*DRD4*) *C-*521T (rs1800955), which has been associated with the ERN and methylenetetrahydrofolate reductase (*MTHFR*) *C677T* (rs1801133), which has been associated with error-related dACC activation. We then compared the effects of each polymorphism on the two error markers modeled as a bivariate response.

**Results:**

We replicated our previous report of a posterior cingulate source of the ERN in healthy participants in the schizophrenia and obsessive-compulsive disorder groups. The effect of genotype on error markers did not differ significantly by diagnostic group. *DRD4 C-521T* allele load had a significant linear effect on ERN amplitude, but not on dACC activation, and this difference was significant. *MTHFR* C677T allele load had a significant linear effect on dACC activation but not ERN amplitude, but the difference in effects on the two error markers was not significant.

**Conclusions:**

*DRD4 C-521T*, but not *MTHFR* C677T, had a significant differential effect on two canonical error markers. Together with the anatomical dissociation between the ERN and error-related dACC activation, these findings suggest that these error markers have different neural and genetic mediation.

## Introduction

Adaptive, flexible behavior depends on the ability to recognize errors and adjust responses to improve outcomes. Deficits in these abilities characterize several neuropsychiatric disorders including schizophrenia, obsessive-compulsive disorder (OCD) and autism spectrum disorder (ASD) and may contribute to maladaptively rigid and repetitive behavior [1]. Accordingly, illuminating the neural and genetic mediation of error processing is important for both basic and clinical neuroscience. Neuroimaging studies have identified two highly reliable neural correlates of errors: the error-related negativity (ERN), an event-related potential that peaks ∼100 ms following an error, and functional MRI (fMRI) activation of the dorsal anterior cingulate cortex (dACC) for erroneous compared with correct responses (see [2]). Although both of these error markers have been extensively characterized, their exact functions and how they are related remain a topic of debate. While influential models postulate that ERN is generated by the dACC [2]–[4], a review of source localization studies and recent evidence instead support a posterior cingulate cortex (PCC) generator of the ERN [5]. Monkey single-unit recordings confirm increased neuronal firing in the PCC after error commission [6]. This anatomical dissociation suggests that error-related dACC activation, rather than being a hemodynamic reflection of the ERN, indexes a different process. Here, we tested the hypothesis that dACC activation and the ERN also have different genetic mediation, which, if confirmed, would further the evidence of distinct underlying mechanisms. We measured both error markers in the same individuals performing the same task and examined the contributions of two single nucleotide polymorphisms (SNPs): dopamine D4 receptor (*DRD4*) *C-521T* (rs1800955), which has been associated with the ERN [7] and methylenetetrahydrofolate reductase (*MTHFR*) *C677T* (rs1801133), which has been associated with dACC activation [8], [9]. No study has compared their influence on both phenotypes.

Converging lines of evidence support a role for dopamine (DA) in error processing [4]. ERN amplitude shows strong heritability among twin pairs [10] and several DA-related genetic polymorphisms have been variably associated with the ERN (for review see [11]). These include *DRD2-TAQ-1A* [12] but see [13] for a negative result; *COMT Val^158^Met* [14] but see [15]; *DAT1 3′-UTR* variable number of tandem repeats (VNTR) [12] but see [16], [17]; and *DRD4 exon 3 VNTR* [17]. The present study examined *DRD4 C-521T*, a SNP in the promoter region of the gene encoding the DA D4 receptor protein, based on evidence of its association with schizophrenia [18], [19], [20] and the observation that *T*-homozygotes have a larger ERN amplitude than *C*-homozygotes [7].

Two prior studies from our group have examined the effects of *MTHFR C677T* on error-related dACC activation [8], [9]. The hypofunctional *677T* allele was associated with reduced error-related dACC activation in three independent samples, one comprising healthy individuals and the other two comprising schizophrenia patients. *MTHFR C677T* may influence several steps in the DA lifecycle by regulating methylation reactions. Each copy of the *677T* allele reduces MTHFR activity by 35% [21] and in two samples, error-related dACC activation was linearly related to the number of *677T* alleles [9]. The *677T* allele is also associated with increased risk for schizophrenia [20], and with increased severity of negative symptoms [22], worse executive function [23] and reduced dorsolateral prefrontal activation during working memory performance [24] in patients with schizophrenia.

We investigated the hypothesis of a double dissociation in genetic mediation of these error markers. We expected each *MTHFR C677T* allele to reduce error-related dACC activation but not affect the ERN, and each *DRD4 C-521T* allele to increase ERN amplitude, but not affect dACC activation. Participants performed an antisaccade paradigm during both fMRI and simultaneous electroencephalography (EEG) and magnetoencephalography (MEG). Antisaccades require inhibition of the prepotent response of looking toward a suddenly appearing stimulus and the substitution of a gaze in the opposite direction. Antisaccade errors (i.e., looking toward the stimulus) reliably elicit both dACC activation [25], [26] and the ERN [27], [28], [29]. We compared the effects of each polymorphism on the two error markers modeled as a bivariate response, and also examined the source of the ERN using anatomically-constrained EEG/MEG.

## Methods

### Participants

A total of 144 participants enrolled in a clinical study of error processing. Of these, 105 completed both the fMRI and EEG sessions and 13 were excluded for not having at least 10 usable error trials in each modality. The final sample of 92 (62 male; age 36±13 years) comprised 33 healthy participants, 28 participants diagnosed with schizophrenia, 18 with OCD and 13 with ASD. Fifty of these participants (23 healthy; 27 schizophrenia) were included in a previous analysis of *MTHFR C677T* effects on error-related dACC activation [9]. Participants in each group were divided by *MTHFR C677T* and *DRD4 C-521T* genotype (Table 1).

**Table 1:** Breakdown of study sample by allele load for each SNP.

	*MTHFR C677T*			*DRD4 C-521T*		
	C/C (0)	C/T (1)	T/T (2)	T/T (0)	C/T (1)	C/C (2)
Healthy participants	16 (41%)	13 (32%)	4 (31%)	10 (37%)	15 (32%)	8 (44%)
Schizophrenia	12 (31%)	13 (32%)	3 (23%)	9 (33%)	14 (30%)	5 (28%)
OCD	7 (18%)	7 (18%)	4 (31%)	4 (15%)	11 (23%)	3 (17%)
ASD	4 (10%)	7 (18%)	2 (15%)	4 (15%)	7 (15%)	2 (11%)
**Totals**	39	40	13	27	47	18

Allele load (0,1,2) refers to the number of risk alleles: *677T* for *MTHFR C677T* and *521C* for *DRD4 C-521T.*

Healthy participants were screened to exclude a personal history of neurological or psychiatric disorder (SCID-Non-patient edition) [30] and a family history of anxiety disorder, OCD, schizophrenia spectrum disorder or ASD. Clinical diagnoses of schizophrenia and OCD were confirmed by medical records review and the Structural Clinical Interview for DSM-IV (SCID) [31]. OCD participants were also required to have a Yale-Brown Obsessive Compulsive Scale (Y-BOCS) [32], [33] total score >16. Clinical diagnoses of ASD were confirmed with the Autism Diagnostic Interview-Revised [34] and the Autism Diagnostic Observation Schedule Module 4 [35] administered by research personnel with established reliability. Patients were all either unmedicated or on stable doses of medication for at least eight weeks. All participants were screened to exclude substance abuse or dependence within the preceding six months and any independent condition that might affect brain function. Participants gave written informed consent and the protocol was approved by the Partners Human Research Committee.

### Genotyping

A saliva sample was acquired with an Oragene self-collection kit (DNA Genotek, Ottawa). *MTHFR C677T* and *DRD4 C-521T* genotyping used allele-specific probes in an assay combining Polymerase chain reaction (PCR) and the 5′ nuclease (Taqman) technique. The specific primers and probes for *MTHFR C677T* were based on published data [23] and synthesized by Applied Biosystems. PCR for DRD4 C-521T genotyping was performed in a 9.0 ul PCR reaction that contained 15 ng of DNA, 1X PCR Buffer, 11% DMSO, 0.55 uMol each dATP, dCTP and dTTP, 0.27 uMol dGTP, 0.55 uMol 7-deaza-2′deoxyguanosine 5′-triphosphate, 2.5 mM MgCl_2_, 2.5 pmol of forward (labeled) and reverse primer (5′-GACCGCGACTACGTGGTCTACTC-3′ and 5′-CTCAGGACAGGAACCCACCGAC-3′), and 0.5 U Amplitaq Gold. The thermocycling conditions consisted of initial denaturation for 15 mins at 95°C, 35 cycles of denaturation at 94°C for 30 seconds, annealing 66°C for 30 seconds and extension at 72°C for 45 seconds with a final extension at 72°C for 10 minutes.

### Multi-Dimensional Scaling Analysis

To control for population stratification, a subset of the analyses were restricted to a Caucasian-only sample. This group was defined based upon both self-report and a multi-dimensional scaling analysis (MDS) performed using an ancestry informative marker set (AIMs) of SNPs. The AIMs panel contains a set of markers that best differentiate and cluster individuals in a dataset into continental populations. Multi-dimensional scaling (MDS) analysis was performed in PLINK (population-based linkage; http://pngu.mgh.harvard.edu/~purcell/plink/0 [36] combining the HapMap Phase 3 (HapMap3) data set with this dataset in order to visualize sample clustering by race/ethnicity in a two-dimensional scatter plot and help assist in measuring genetic distance. In MDS analysis, PLINK assigns an Identity by State (IBS) score for each sample pair at each marker. Using these IBS scores, PLINK performs an algorithm to reduce the IBS information to fewer dimensions. We created a scatter plot using the first two dimensions or axes of variation to determine where the samples in this study fell relative to the HapMap3 samples and then compared those results to the self-reported racial/ethnicity data. Of the 92 samples, 74 samples self-reported Caucasian and also fell within the HapMap3 European/Caucasian cluster.

### Antisaccade paradigm

**Figure 1:**
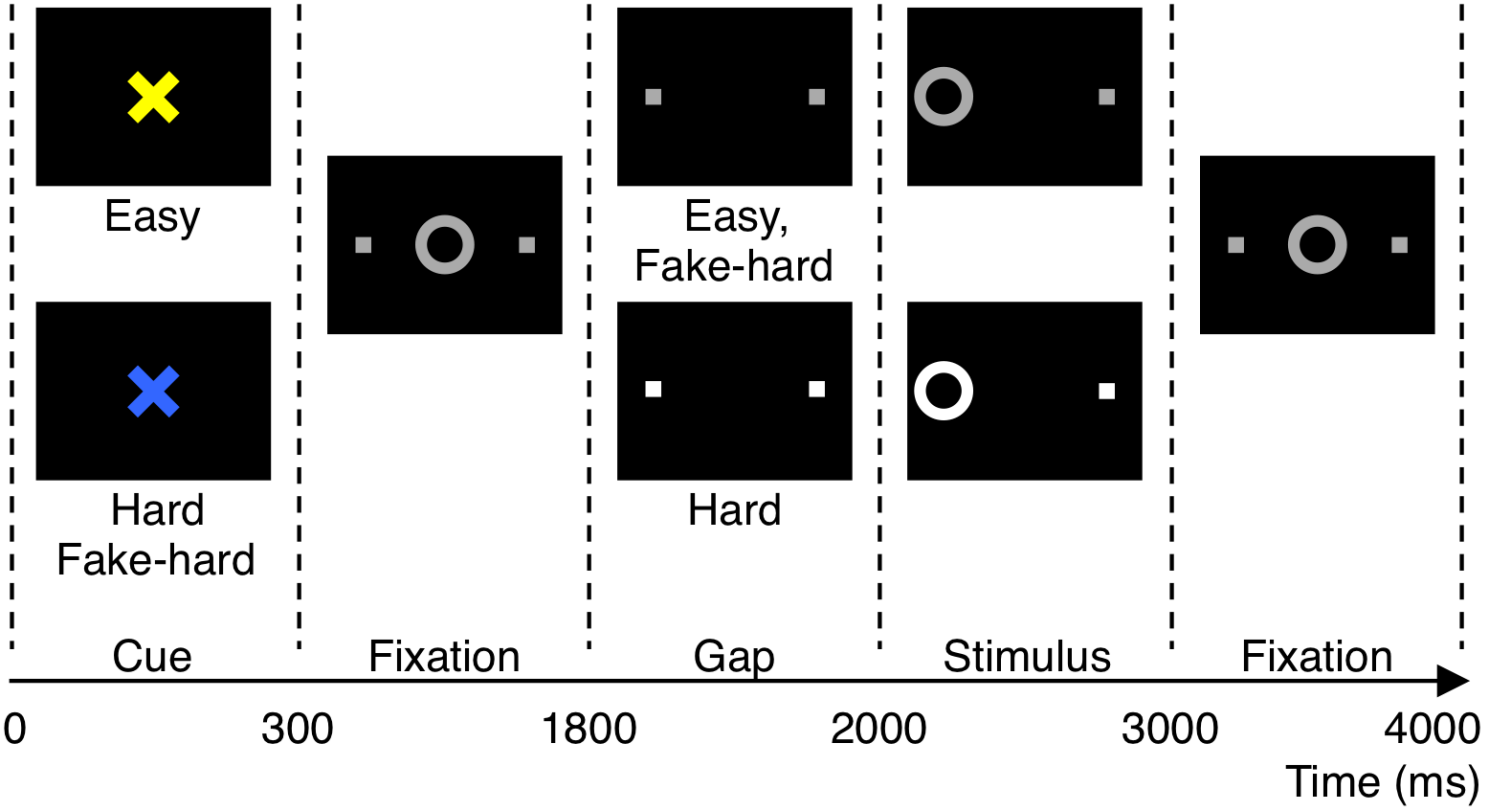
Antisaccade paradigm. Schematic and timeline of the three conditions: easy, hard, and fake-hard. Each trial lasted 4 s and began with an instructional cue (300 ms), either a blue or yellow “X” that indicated whether the trial was hard or easy. The mapping of cue color to trial type was counterbalanced across participants. The cue was horizontally flanked by two white squares of 0.4° width that marked the potential locations of stimulus appearance, 10° left and right of center. The squares remained visible for the duration of each run. At 300 ms, the instructional cue was replaced by a white fixation ring of 1.3° diameter at the center of the screen. At 1800 ms, the fixation ring disappeared (200 ms gap). At 2000 ms, the fixation ring reappeared at one of the two stimulus locations, right or left with equal probability. This was the imperative stimulus to which the participant responded by making a saccade in the opposite direction. The ring remained in the peripheral location for 1000 ms and then returned to the center, where participants were instructed to return their gaze for 1000 ms before the start of the next trial. Fixation epochs were simply a continuation of this fixation display. Hard trials were distinguished by a 3 db increase in luminance of the peripheral squares starting during the gap. Except for the hard cue, fake-hard trials were identical to easy trials.

The antisaccade paradigm (Fig. 1) was programmed in Matlab Psychtoolbox (Mathworks, Natick, MA), and consisted of three types of antisaccade trials: Hard (40%), Easy (50%), and Fake-Hard (10%). Hard trials introduced a distraction during the gap – a 3 dB luminance increase of the peripheral squares that mark the location of stimulus appearance. Fake-Hard trials started with a cue indicating a hard trial, but were otherwise identical to Easy trials (i.e., there was no luminance change). They were included as a control condition to allow an examination of the effects of a hard vs. easy cue on fMRI activation unconfounded by the change in luminance that characterizes hard trials. In the present study, error and correct trials were combined across all three trial types for analysis.

Antisaccade trials were balanced for right and left stimuli. Randomly interleaved with the saccadic trials were fixation epochs lasting 2, 4, or 6 s, which provided a baseline and introduced “temporal jitter” to optimize the analysis of rapid presentation event-related fMRI data [37]–[39]. The schedule of events was determined using a technique to optimize the statistical efficiency of event-related designs [40]. Each task run lasted 5 min 16 s and generated an average of 64 antisaccade trials and 20 fixation epochs. Participants performed six runs in fMRI and eight runs in EEG/MEG. The order of fMRI and EEG/MEG sessions was counterbalanced.

Prior to the first scanning session, participants practiced in a mock MRI scanner, were encouraged to respond as quickly and accurately as possible, and were told that in addition to the base rate of pay, they would receive 5¢ for each correct response.

### Recording and scoring of eye movement data

The ISCAN fMRI Remote Eye Tracking Laboratory (ISCAN, Burlington, MA) recorded eye position during fMRI using a 120 Hz video camera. During EEG, eye movements were monitored using two pairs of bipolar EOG electrodes, one vertical (above and below the left eye) and one horizontal. Horizontal EOG activity recorded during a brief calibration allowed an estimate of gaze position for scoring antisaccades [29], [41].

Eye movement data were scored in MATLAB (Mathworks, Natick, MA) using a partially automated program. Saccades were identified as horizontal eye movements with velocities exceeding 47°/s. The onset of a saccade was defined as the point at which the velocity of the eye first exceeded 31°/s. Trials with initial saccades in the direction of the stimulus were scored as errors. Reaction time (RT) was defined as the onset time of the initial saccade relative to the appearance of the stimulus. Error rates were logit-transformed before analysis to normalize their distribution. Group differences in error rates and saccadic RT on correct trials were assessed with ANOVA. Since error rates were similar in the EEG and fMRI sessions, they were averaged across modalities for further analysis.

### MRI acquisition

Images were acquired with a 3T Siemens Trio whole body high-speed imaging device (Siemens Medical Systems, Erlangen, Germany), equipped for echo planar imaging (EPI). Eighty-two participants were scanned with a 12-channel head coil and 10 healthy participants with a 32-channel head coil. A high-resolution structural scan was acquired in the sagittal plane using 3D rf-spoiled magnetization prepared rapid gradient echo (MP-RAGE) sequences (12-channel: TR/TE/Flip = 2530 ms/3.39 ms/7°; FOV = 256 mm, 176 1.33×1×1.33 mm in-plane slices; 32-channel: TR/TE/Flip = 2530 ms/1.61+1.78 *n*, *n* = 0–3/7°; iPAT = 3; FOV = 256 mm, 176 1×1×1 mm in-plane slices). To construct the boundary-element model surface for each participant’s MEG/EEG source estimation, we acquired a multi-echo multi flip angle (5°) fast low-angle shot (FLASH) pulse sequence (610 Hz/pixel, TR = 20 ms, TE = (1.89+2 *n*) ms, *n* = 0–7, 128 1×1.33 mm in-plane sagittal slices, 1.33 mm thickness).

Functional images were acquired using a gradient echo T2* weighted sequence (12-channel: TR/TE/Flip = 2000 ms/30 ms/90°, 32 contiguous horizontal slices parallel to the inter-commissural plane, voxel size: 3.1×3.1×3.7 mm, interleaved; 32-channel: TR/TE/Flip = 2000 ms/28 ms/77°, iPAT = 3, 41 contiguous horizontal slices parallel to the inter-commissural plane, voxel size: 3.1×3.1×3.1 mm, interleaved). The functional sequences included prospective acquisition correction (PACE) for head motion [42].

### fMRI Analysis

Analyses were conducted on each participant’s inflated cortical surfaces reconstructed from the MP-RAGE scan using FreeSurfer (http://surfer.nmr.mgh.harvard.edu) segmentation, surface reconstruction, and inflation algorithms [43], [44]. Functional and structural scans were spatially normalized to a template brain consisting of the averaged cortical surface of an independent sample of 40 adults (Buckner laboratory, Washington University, St. Louis, MO) using Freesurfer’s surface-based spherical coordinate system, which employs a non-rigid alignment algorithm that explicitly aligns cortical folding patterns and is relatively robust to inter-individual differences in the gyral and sulcal anatomy of the cingulate cortex. Cortical activation was localized using automated surface-based parcellation software [45]. To facilitate comparison with other studies, approximate Talairach coordinates were derived by mapping surface-based coordinates back to the original structural volume for each of the individuals whose brains were used to create the template brain, registering the volumes to the Montreal Neurological Institute (MNI305) atlas [46] and averaging the corresponding MNI305 coordinates. These coordinates were transformed to standard Talairach space (http://imaging.mrc-cbu.cam.ac.uk/imaging/MniTalairach).

In addition to prospective motion correction (PACE), functional scans were retrospectively corrected for motion using the AFNI algorithm [47], intensity normalized, and smoothed using a 3D 8 mm FWHM Gaussian kernel. Functional images were aligned to the MP-RAGE scan for each participant.

Finite impulse response (FIR) estimates [38], [39] of the event-related hemodynamic responses were calculated for error and correct trials for each participant. This involved using a linear model to provide unbiased estimates of the average signal intensity at each time point without making *a priori* assumptions about the shape of the hemodynamic response. Estimates were computed at 12 time points with an interval of 2 s (corresponding to the TR) ranging from 4 s prior to the start of a trial to 18 s after the start. Temporal correlations in the noise were accounted for by prewhitening using a global estimate of the residual error autocorrelation function truncated at 30 s [39].

The dACC was defined using automated surface-based parcellation software [45] that delineated cingulate cortex, which was then divided into dACC, rACC, and PCC [48]. Using this anatomical definition, error-related dACC activation was measured at the maximal vertex in each hemisphere for each participant in the error vs. correct contrast at 6 s, the time of maximal error-related activation in the group and in a prior antisaccade study [26]. Because error-related activation in the left and right dACC was strongly correlated (r = .89) we averaged activation across the hemispheres to simplify the model.

### MEG/EEG Acquisition and Analysis

EEG and MEG ware acquired simultaneously in a magnetically shielded room (IMEDCO, Hagendorf, Switzerland). MEG was recorded using a dc-SQUID Neuromag VectorView system (Elekta-Neuromag, Helsinki, Finland) comprising 306 sensors arranged in triplets of two orthogonal planar gradiometers and a magnetometer, distributed at 102 locations around the entire scalp. EEG was recorded using a 70-channel electrode cap. Electrode impedances were brought below 20 KOhm at the start of each recording session. All signals were identically filtered to 0.1–200 Hz bandpass and digitized at 600 Hz.

To allow registration of EEG/MEG and MRI data and to record head position relative to the sensor array, the locations of three fiduciary points (nasion and auricular points) defining a head-based coordinate system, the sites of four head position indicator (HPI) coils, and a set of points from the head surface were digitized using a 3 Space Fastrak digitizer (Polhemus, Colchester, VT, USA) integrated with the Vectorview system. At the beginning of each MEG acquisition, currents were fed to the HPI coils and their magnetic fields were used to calculate the relative location of the head with respect to the MEG sensor array.

After excluding noisy EEG channels by visual inspection of the raw data, EEG data were re-referenced to the grand average. MEG channels were processed using the signal-space separation method [49]. Each participant’s continuous MEG and EEG data were low-pass filtered at 40 Hz. Trials with eye blinks were defined by a difference between the maximum and minimum voltage of 150 µV or greater at the vertical EOG channel and excluded from analysis. EEG data, time-locked to the onset of the saccade, were baseline-corrected by subtracting the mean signal during the 100 ms preceding the saccade from the 500 ms that followed the saccade. Data for each of trial type (correct and error) were averaged for each participant.

The ERN was derived using the average signal across the following 10/20 locations: FC1, FCz, FC2, C1, Cz, C2, CP1, CPz, CP2 for each participant. An average was used so as not to exclude participants with bad channels. The peak ERN for the entire sample was identified within the 200 ms following saccadic initiation as the time point of maximal difference for the error vs. correct waveforms (140 ms). The peak ERN for each participant was identified as the point of maximal difference within 50 ms on either side of the group peak [5].

MNE software (www.martinos.org/martinos/userInfo/data/sofMNE.php) was used to derive current source estimates of the difference waveform (error-correct) from the combined EEG and MEG group data. The reconstructed cortical surfaces for each participant, which comprised approximately 100,000 vertices per hemisphere, were decimated to a subset of approximately 3,000 dipole locations (vertices) per hemisphere. The forward solution was calculated using a three-compartment boundary-element model [50] with the inner and outer skull surfaces and the scalp surface segmented from the FLASH images. The head position information from the start of each run was used in the calculation of a forward solution for each run, which were averaged together. The amplitudes of the dipoles at each cortical location were estimated every 4 ms using the anatomically constrained linear estimation approach [51]. The orientations of the dipoles were tightly constrained to the cortical normal direction by setting source variances for the transverse current components to be 0.1 times the variance of the currents normal to the cortical surface [52]. Individual source estimate data were mapped to the template cortical surface. This resulted in a set of source estimates at each time point that were spatially aligned across participants.

To localize the ERN source, we used the source estimate of the difference waveform at the time of the peak ERN for each participant. We employed a *t*-test in each diagnostic group to determine whether the averaged amplitudes of the source estimates differed from zero at each vertex on the cortical surface. Correction for multiple comparisons was based on a permutation analysis, which approximated the null distribution (i.e., no difference between correct and error trials) by randomly swapping the error and correct conditions for each participant (i.e., by multiplying each individual source estimate by either 1 or −1). This procedure was repeated 10,000 times. We then measured the area of the largest cluster of vertices with a significant non-zero current estimate (p≤.05) in each permuted dataset, resulting in a distribution of cluster sizes. This null distribution was then used to determine the probability that the observed cluster size would occur by chance.

### Analysis of genotype effects

We assessed the effects of genotype on our primary outcome variables, dACC activation and ERN amplitude, using linear regressions with allele load (0, 1 or 2) as a covariate for each SNP. Because our hypotheses were directional (i.e., reduced error markers with larger risk-allele load), we conducted one-tailed tests. We also examined the effects of genotype on error rate and on correct trial RTs.

To test the hypothesis that each genotype had significantly different effects on dACC activation and the ERN (i.e., the magnitude of activation and ERN amplitude had different slopes as a function of allele load) we modeled the two error markers as a bivariate response and employed multivariate regression analyses using the “R” statistical computing environment [53]. To consider the error markers as a bivariate response it was necessary to standardize each measure (i.e., to have a zero mean and a standard deviation of 1) since they have different units of measurement. Allele load (0, 1, 2) refers to the number of *677T* alleles for *MTHFR C677T* and the number of -*521C* alleles for *DRD4 C-521T* and was treated as a linear covariate. The effect of each allele load is described by four slopes (two SNPs x two error markers) and the differences between slopes were tested using one-tailed tests to reflect our *a priori* hypotheses.

Secondary analyses considered models with diagnosis and its interaction with genotype as covariates, excluded non-Caucasians, and used a dominant model of allele load (*677T* carriers vs. C homozygotes and 521C carriers vs. T homozygotes). We assessed the effect of the interaction of diagnosis with genotype on response by comparing models with and without covariates for diagnosis using ANOVA.

## Results

### Antisaccade performance

As error rates did not differ significantly in fMRI and MEG/EEG (t(90) = −1.20, p = .23), the results are averaged across modalities (Table 2). The overall antisaccade error rate was 20±16% (mean ± SD) and almost all errors were self-corrected (96%). Error rates differed by diagnosis (F(3,88) = 5.26, p = .002) reflecting that participants with schizophrenia made more errors than healthy (t(59) = 3.42, p = .001) and OCD participants (t(44) = 2.72, p = .009). Error rates were associated (trend) with allele load for *MTHFR C677T* (p = .07; Table 3), but not for *DRD4 C-521T* (p = .80). When diagnosis was included as a factor in the model, the relation of error rate with allele load became significant for *MTHFR C677T* (F(1,87) = 4.14, p = .045) but not *DRD4 C-521T*. The interaction of *MTHFR C677T* with diagnosis was not significant (F(3,84) = 0.43, p = .73) indicating that diagnosis did not substantially affect the results.

**Table 2:** Outcome measures divided by diagnosis.

	HC (n = 33)	SZ (n = 28)	OCD (n = 18)	ASD (n = 13)	Combined (n = 92)
Error rate (%)^1^	16±10	30±19	16±8	20±11	21±15
Error-related dACC activation (% change)	.11±.07	.13±.10	.10±.07	.15±.13	.12±.09
ERN (µV)	3.7±1.6	2.4±2.1	4.2±2.7	2.7±1.5	3.3±2.1

1 Collapsed across fMRI and EEG sessions. Note that participants with fewer than 10 usable error trials per modality were excluded from the study.

**Table 3:** Results of the univariate analyses examining the effect of each SNP on each outcome measure.

	*C/C*	*C/T*	*T/T*	*Regression Result*
	*MTHFR C677T*			
Error rate (%)	18±13	22±16	24±15	t(90) = 1.85 p = .07^1^
dACC activation (% change)	.10±.10	.04±.09	.04±.08	t(90) = −1.75, p = .04*
ERN (µV)	2.6±2.0	2.2±2.1	2.2±2.9	t(90) = −0.74, p = .23
	***DRD4 C-521T***			
Error rate (%)	20±11	21±15	22±19	t(90) = −0.26; p = .80
dACC activation (% change)	.08±.08	.07±.10	.06±.11	t(90) = 1.04, p = .85
ERN (µV)	3.3±1.9	2.1±2.1	1.8±2.5	t(90) = −1.75, p = .04*

1 When diagnosis was included as a factor in the model this effect became significant (p = .045).

*significant at p≤.05.

### Error-related dACC Activation

**Figure 2:**
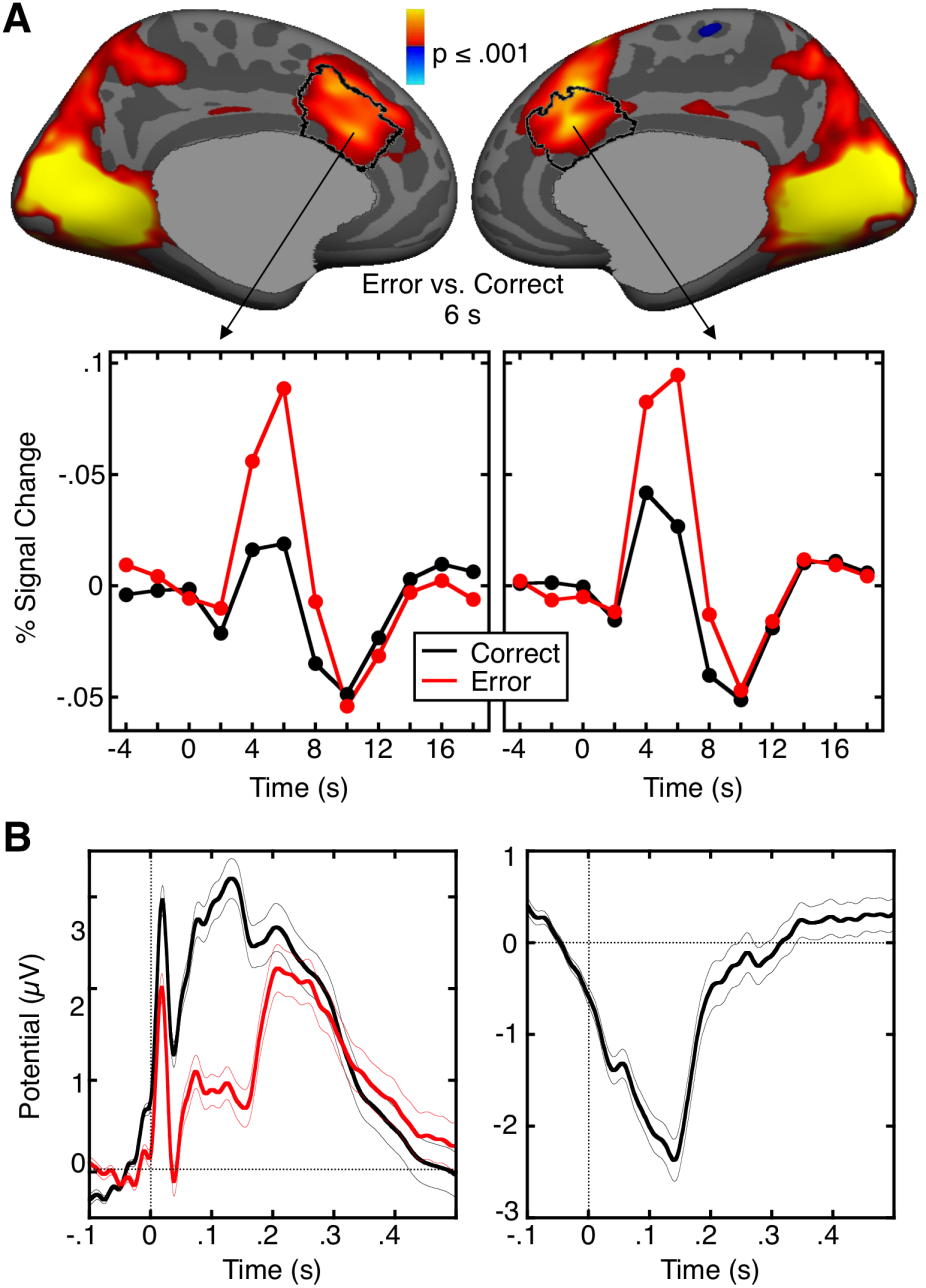
fMRI and EEG error markers. A. Error-related dACC activation. Statistical maps of activation at 6 s in the contrast of error vs. correct are displayed on the inflated medial cortical surfaces. The dACC ROI is outlined in black. Warm colors indicate stronger activation on errors. The gray masks cover subcortical regions in which activity is displaced in a surface rendering. Line graphs show hemodynamic response functions for correct and error trials in the vertices with maximal error-related activation in the dACC. B. The ERN. The left panel shows grand average waveforms for correct (black) and error (red) trials, time locked to the onset of the saccade. The right panel shows the difference waveform, obtained by subtracting the correct waveform from the error waveform. The thin lines on either side of the waveforms represent the standard error of the mean at each time point.

**Figure 3:**
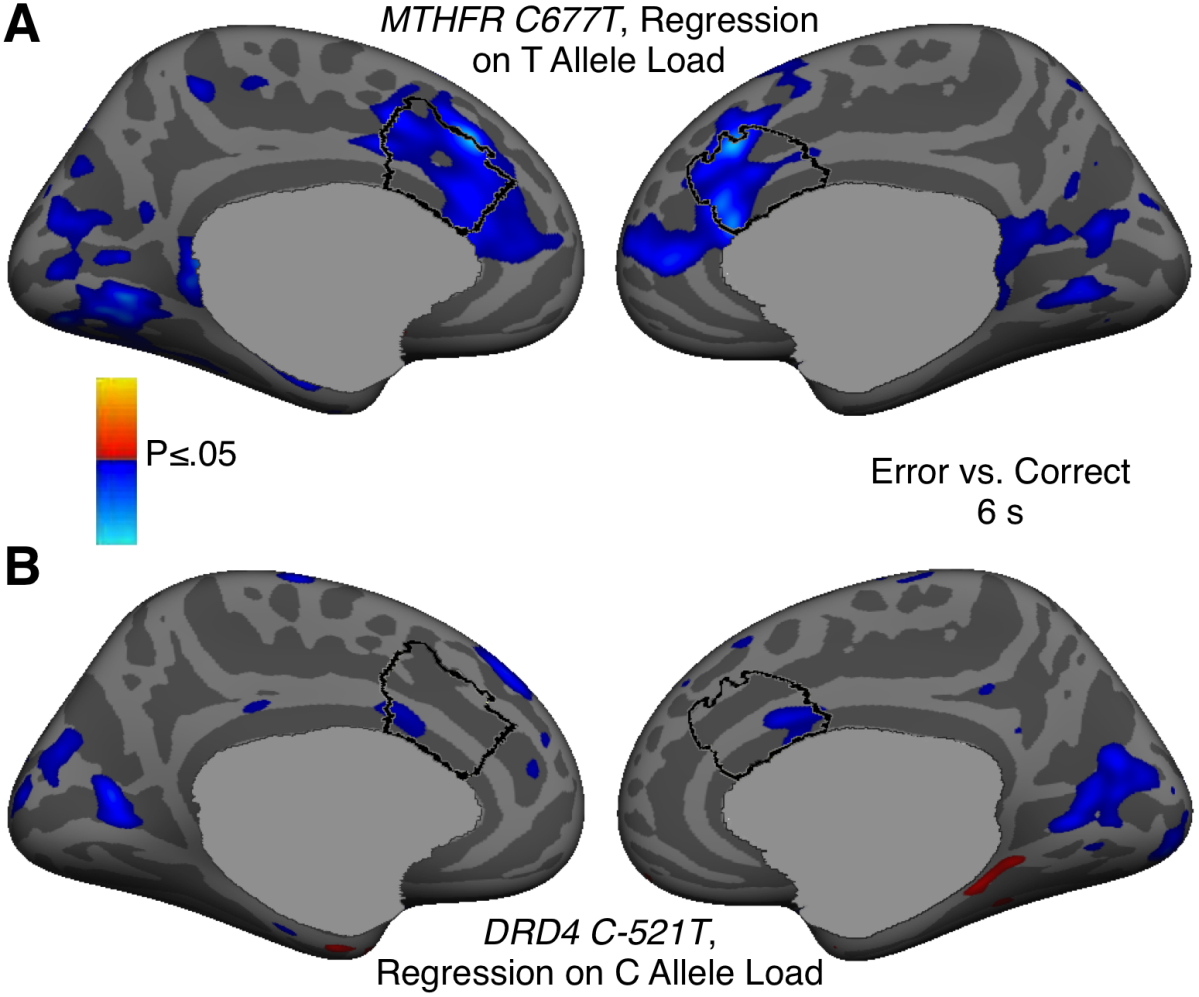
Genetic modulation of error-related dACC activation. A: *MTHFR C677T*. B: *DRD4 C-521T*. Statistical maps show regressions of activation in the error vs. correct contrast on allele load. Blue colors represent a negative correlation, i.e., stronger activation associated with more 677T (A) or -521C (B) alleles. The gray masks cover subcortical regions in which activity is displaced in a surface rendering.

Relative to correct trials, errors were associated with increased dACC activation (Fig. 2A; Talairach locations of maximal activation: left x = −7, y = 24, z = 23 and right 9, 23, 25), which did not differ by diagnostic group (F(3,88) = 1.03, p = .39, Table 2). Error-related dACC activation was associated with *MTHFR C677T* but not *DRD4 C-521T* allele load (Fig. 3, Table 3). When diagnosis and its interaction with allele load was added to the models, the interaction was not significant for either *MTHFR C677T* (p = .72) or *DRD4 C-521T* (p = .14), indicating that diagnosis did not substantially affect the results.

### ERN

**Figure 4:**
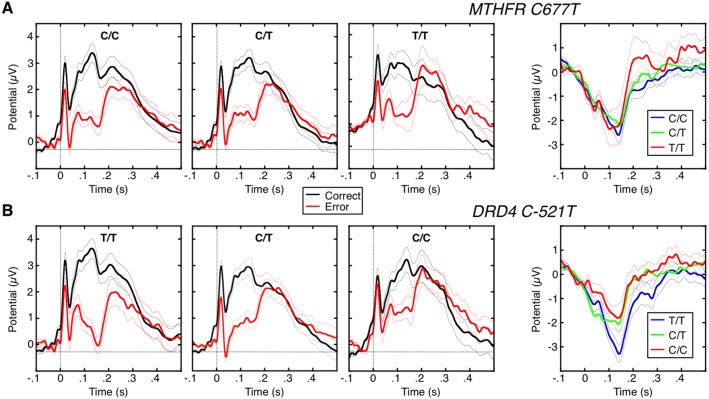
Genetic modulation of the ERN. A: *MTHFR* C677T. B: *DRD4 C-*521T. Correct and error trial waveforms are shown for every allele combination of each polymorphism. The error-correct difference waveforms for each allele combination is shown on the right column. The thin lines on either side of the waveforms represent the standard error of the mean at each time point.

The group ERN was observed as a robust negative deflection in the difference waveform for error vs. correct trials that peaked 140 ms after the saccadic response (Fig. 2B). The ERN differed by diagnosis (F(3,88) = 4.19, p = .008, Table 2). Post-hoc t-tests indicated that schizophrenia participants had a smaller amplitude ERN than healthy (t(59) = 2.89, p = .005) and OCD (t(44) = 2.62, p = .01) participants. *DRD4 C-521T* was significantly associated with the amplitude of the ERN (t(90) = −1.75, p = .04, Fig. 4, Table 3), but *MTHFR C677T* was not (t(90) = −0.74, p = .23). When divided by diagnosis, the association with *DRD4 C-521T* allele load reached significance in the schizophrenia group (t(26) = −1.66, p = .05), and approached significance in healthy participants (t(31) = −1.34, p = .09). When diagnosis and its interaction with allele load was added to the models, the interaction was not significant for either *DRD4 C-521T* (p = .92) or *MTHFR C677T* (p = .53) indicating that diagnosis did not substantially affect the results.

Our prior finding of a PCC source for the ERN in 30 of the present 33 healthy participants [5] was replicated in the schizophrenia and OCD groups (Fig. S1, Table S1). In both groups, there was a significant cluster of dipole sources in the PCC bilaterally. The PCC cluster in the smaller ASD group did not reach significance, but the source localization was similar to the other groups.

### Bivariate Analyses

**Figure 5:**
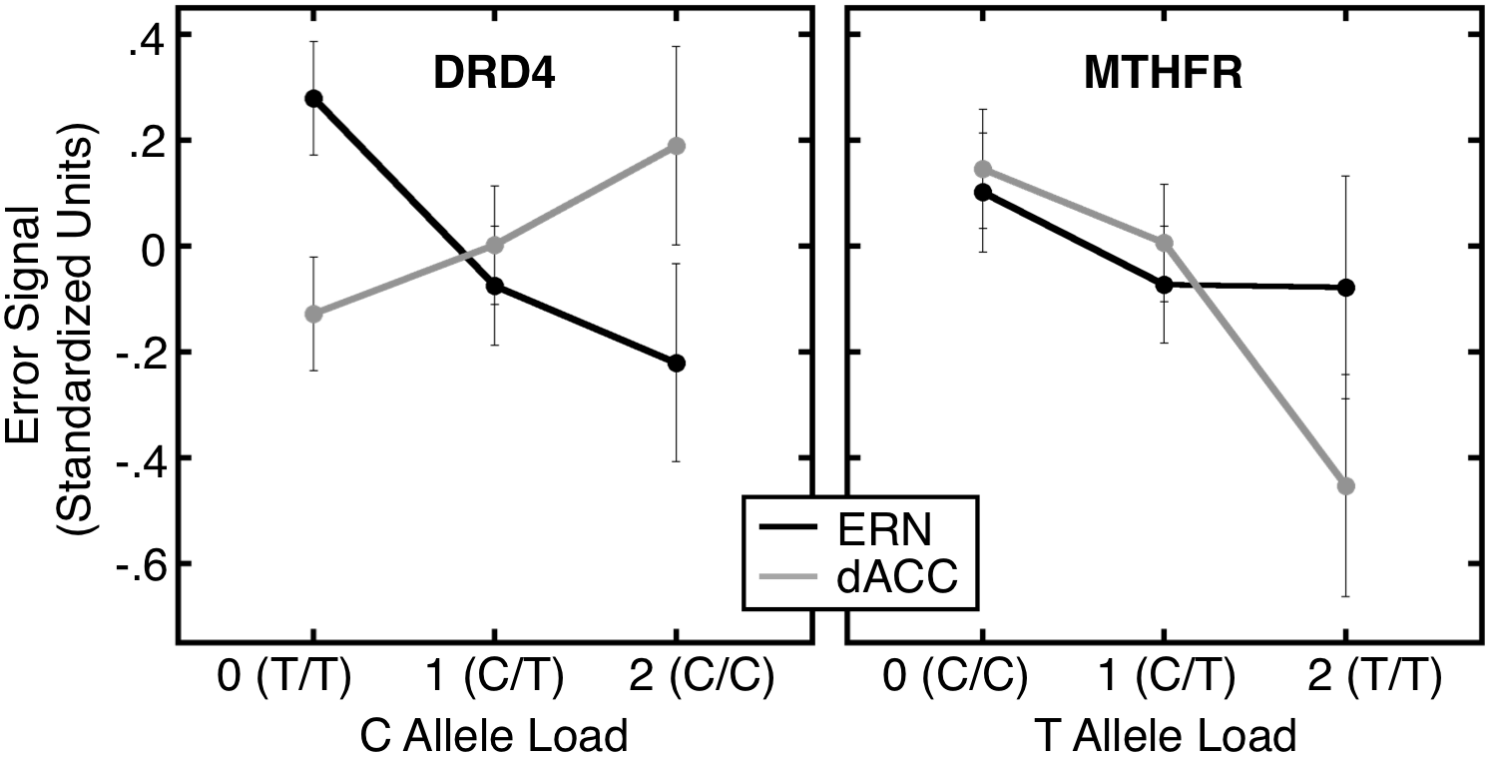
Genetic dissociation between error-related dACC activation and the ERN. Both error markers are shown in standardized units as a function of risk allele load (677T for *MTHFR C677T*, -521C for *DRD4 C-521T*). Error bars represent within subject confidence intervals [75] for each allele combination.

These analyses tested for differential effects of genotype on error markers. The results were similar in the primary model, which included the entire group and a linear effect of allele load, and in the other models that included either the entire group or Caucasians only, did or did not include diagnosis as a covariate, or did or did not use a dominant model of allele load (Fig. 5, Table 4).

**Table 4:** Results of the bivariate analyses testing the differential effects of each SNP on error markers.

	*MTHFR C677T*		*DRD4 C-521T*	
	Diagnosis as covariate?		Diagnosis as covariate?	
	no	Yes	no	yes
Entire sample (n = 92)	t(89) = 0.83 p = .21	t(86) = 0.92 p = .26	t(89) = 2.05 p = .02*	t(86) = 2.43 p = .01*
Caucasians only (n = 74)	t(71) = 0.89 p = .19	t(68) = 1.25 p = .11	t(71) = 1.92 p = .03*	t(68) = 2.30 p = .01*
Whole sample, dominant model	t(89) = 0.49 p = .31	t(86) = .75 p = .23	t(89) = 1.86 p = .03*	t(86) = 2.23 p = .01*

The primary analysis included the entire sample, allele load, and no covariate for diagnosis.

*significant at p≤.05.

Regardless of the model used, *DRD4 C-521T* genotype had a significantly stronger effect on the ERN than on error-related dACC activation. The interactions of diagnosis with genotype were not significant in any of the four models (i.e., dominant/non-dominant, all data/Caucasians only; all p’s ≥.19) suggesting the effects were similar across diagnostic groups.

For *MTHFR C677T,* the difference in the effect of allele load on the two error markers did not reach significance in any model. Nor were the interactions of diagnosis with genotype significant in any of the four models (p’s ≥.11).

## Discussion

We tested the hypothesis that two canonical neural markers of errors, the ERN and error-related dACC activation have distinct genetic mediation. We previously reported a PCC source for the ERN in healthy individuals [5] and now, using identical anatomically-constrained EEG/MEG source localization methods, we have replicated this finding in schizophrenia and OCD. This reinforces the anatomical dissociation between error-related dACC activation and the ERN. We now also report evidence of different genetic mediation. First, we replicated the finding that *DRD4 C-521T* is associated with increased ERN amplitude [7], here in a linear model of allele load. This effect was significantly greater than the *DRD4 C-521T* effect on dACC activation, which was not significant. In contrast, we did not find a significant differential effect of *MTHFR C677T* on error markers. *MTHFR C677T* was associated with blunted error-related dACC activation, as previously reported in an independent sample [8] and in subset of the present sample [9]. Although *MTHFR C677T* did not significantly affect ERN amplitude, the difference in the slopes of the relation of allele load with each error marker was not significant. While these findings support the hypothesis of differential genetic mediation of these error markers (i.e., *DRD4 C-521T* showed a significantly stronger effect on ERN than dACC activation), they do not support the hypothesis of a double-dissociation since *MTHFR C677T* did not show a significantly greater effect on dACC activation than the ERN. Together with the anatomical dissociation between the ERN and error-related dACC activation, these findings suggest that these error markers have different neural and genetic mediation. These findings challenge theories that these two error markers reflect the same underlying neural process measured by different techniques.

This study replicated the finding that *DRD4 C-521T* is associated with increased ERN amplitude [7] and extended it by showing linear effect of allele load. Moreover, the *DRD4 C-521T* effect on ERN amplitude was significant in the schizophrenia group alone, making this the first report of this effect in schizophrenia. There is strong evidence of a role for DA in mediating the ERN. ERN amplitude is affected by pharmacological agents that affect DA [54]–[56] and by Parkinson’s Disease, which is associated with a loss of midbrain DA neurons [57]–[59]. The effect of *DRD4 C-521T* on DA receptor availability is controversial, with one study reporting a 40% decrease in transcriptional efficiency [18], but another finding no effect [60]. In an influential model, the ERN is generated when a mismatch between the intended (correct) versus actual (error) outcome (i.e., prediction error) leads to a phasic decrease in mesencephalic DA release that disinhibits dACC neurons (or, in a revised model, PCC neurons – though DA innervation of the PCC is less than that in the ACC [61], [62]), which give rise to the ERN [4]. If *521T* leads to reduced DA receptor availability, one might expect reduced, not increased, ERN amplitude as is seen with the dopamine antagonist haloperidol [56]. DRD4 knockout mice, however, show increased DA synthesis and turnover in the basal ganglia [63]. While it is possible that the putative reduction in D4 receptor availability in human *521T*-carriers could indirectly lead to stronger error signaling by some compensatory mechanism, the basis of this effect is unknown. Important caveats to the DA theory of ERN generation [4] include that DA is thought to largely play a modulatory or inhibitory role in the cortex, including in the cingulate [64], that its effects lack the temporal precision to generate a phasic error signal and that glutamate, which is thought to be co-released with DA, may instead transmit error signals [65].

Given that the ERN is localized to the PCC, one might ask whether DRD4 also affects error-related fMRI activation of the PCC. As seen in Figure 3B, there was no significant DRD4 effect on PCC activation. This may reflect that there is no compelling fMRI correlate of the ERN in the PCC. As seen in Figure 2A, although there are small clusters of error-related activation in bilateral PCC, they do not survive correction for multiple comparisons despite the sample size of 92. This lack of error-related PCC activation is consistent with most, but not all [66], [67], prior fMRI studies of error processing and may reflect the different sources of fMRI vs. MEG/EEG signals. If the ERN, as has been theorized, arises from disinhibition of cingulate neurons [4], this might not lead to an increase in the BOLD signal [68]. Another possibility is that if the ERN arises due to synchronization of constantly active, but otherwise asynchronous neural populations, this would affect MEG/EEG signals but not necessarily hemodynamic activity. For these reasons, fMRI may not show the ERN.

For *MTHFR C677T*, we previously reported a linear effect of *677T* on error-related dACC activation in a prior analysis of a subset the present healthy and schizophrenia samples [9]. The mechanism of *MTHFR C677T* effects on dACC function is not clear, but in addition to reduced global DNA methylation [69], *677T* may affect the activity of other genes, including those more directly involved in DA function and related to executive function. Consistent with this possibility, *MTHFR C677T* has an epistatic effect with *COMT Val^158^Met*, on dorsolateral prefrontal fMRI activation during working memory performance in schizophrenia [70]. It is possible that *MTHFR 677T* could decrease methylation in the COMT promoter, which could lead to reduced expression of COMT and higher DA availability in the synapse [23].

Despite strong evidence of genetic mediation of neural error markers, there was only weak evidence of genetic mediation of error rate by *MTHFR C677T*. This is not surprising given the limited sample size and that behavior is usually a less sensitive and specific index of genetic effects than brain activity. Behavior may reflect not only the integrity of the brain system of interest, but also of other systems, including the motor output systems that are required to produce the behavior. For example, in the present study, antisaccade error rate is unlikely to be solely determined by the use of errors to improve performance, but may also reflect other factors including inattention, failure to maintain the task set, and failures of response inhibition.

In summary, we report that a genetic polymorphism, previously associated with error processing, differentially modulates neural error markers. The test of differential modulation reached significance for *DRD4 C-521T* but not for *MTHFR C677T*. The lack of a double dissociation may reflect that error-related dACC activation and the ERN are functionally related, although we cannot rule out Type II error given the relatively small sample size. In a previous study, we reported that the dACC region showing error-related activation and the PCC region that was the source of the ERN were functionally connected during antisaccade performance in healthy participants, and also during rest in a separate sample from a large, publically available dataset of resting state fMRI scans [5]. This suggests that the PCC and dACC are constituents of a functional circuit. We previously proposed that the PCC detects errors, giving rise to the ERN and relays this information to the dACC to implement corrective behavior [5]. This was based on our finding that the structural integrity of the cingulum bundle, which connects dACC and PCC [71], predicts the latency to initiate a corrective saccade, as well as other evidence from the literature of a dACC role in behavioral adjustment [72]–[74]. If this model is correct, the strength of the ERN could have downstream effects on error-related dACC activation. Despite evidence of a functional relationship, the present findings support models that view the ERN and error-related dACC activation as anatomically and mechanistically distinct error markers.

## Supporting Information

Figure S1 **Combined EEG/MEG Source estimate of the ERN in each diagnostic group, displayed on the inflated medial cortical surfaces.** The statistical maps show vertices where the current estimate at the time of peak ERN was significantly different from zero. Positive (red) and negative (blue) values indicate currents flowing out and into the cortex, respectively.
(TIF)

Table S1 **ERN source localization based on combined EEG/MEG data.** ERN source localization based on combined EEG/MEG data. Maxima and locations of clusters where dipole sources were significantly different from zero. Clusterwise probabilities (CWP) are based on correction for the entire cortical surface. P-values are provided for the most significant dipole source in each cluster. Current direction in all clusters outwards from the cortical surface.
(DOCX)
