# Supplementary figures and images for: Dissociable Genetic Contributions to Error Processing: A Multimodal Neuroimaging Study
Source: PLoS One. 2014 Jul 10;9(7):e101784. doi: 10.1371/journal.pone.0101784 (PMC4092014; doi:10.1371/journal.pone.0101784)

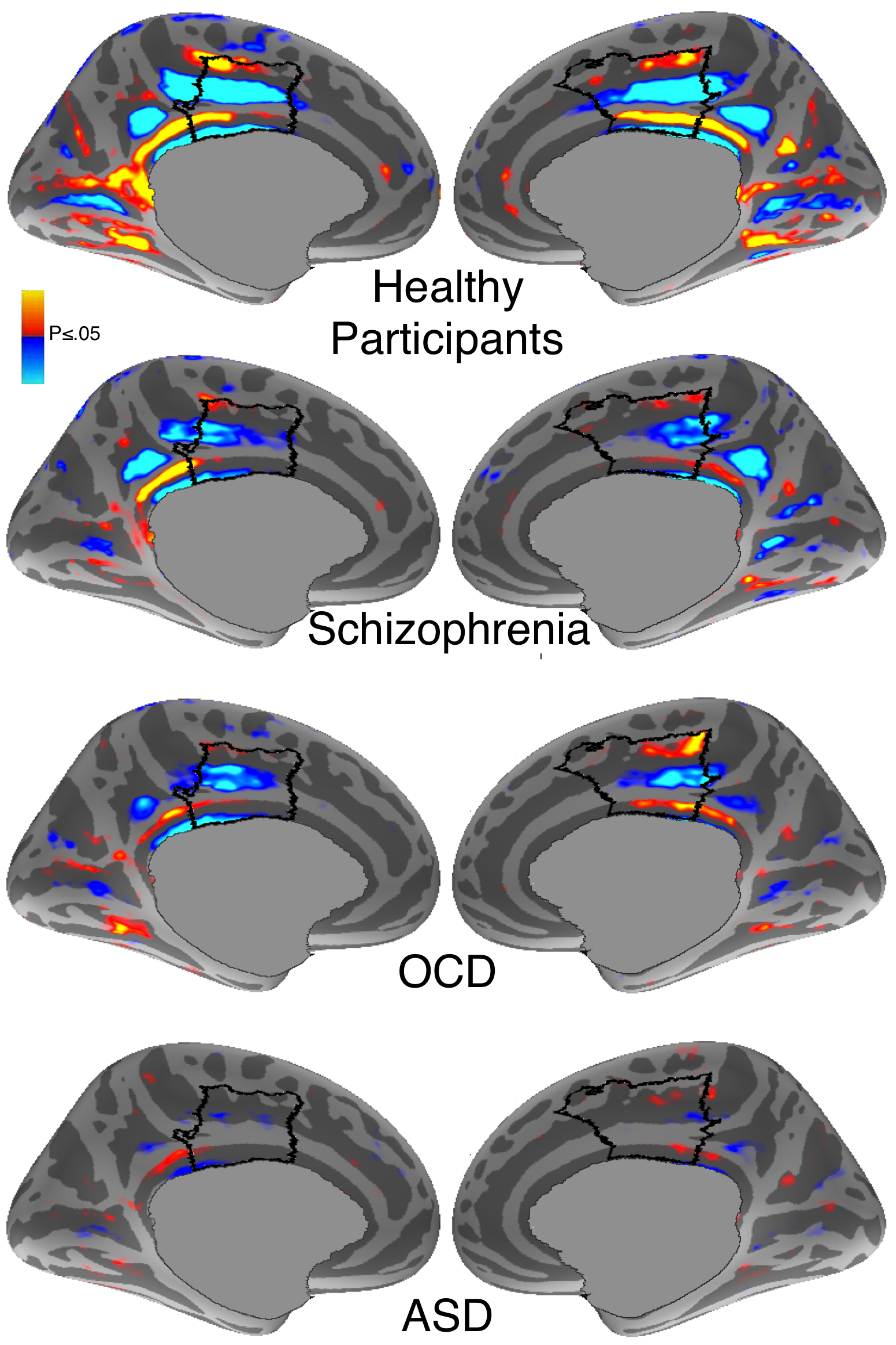

Supplement: Figure S1 — Combined EEG/MEG Source estimate of the ERN in each diagnostic group, displayed on the inflated medial cortical surfaces. The statistical maps show vertices where the current estimate at the time of peak ERN was significantly different from zero. Positive (red) and negative (blue) values indicate currents flowing out and into the cortex, respectively. (TIF) [file pone.0101784.s001.tif]
