# Supplementary material for: Dissociable Genetic Contributions to Error Processing: A Multimodal Neuroimaging Study
Source: PLoS One. 2014 Jul 10;9(7):e101784. doi: 10.1371/journal.pone.0101784 (PMC4092014; doi:10.1371/journal.pone.0101784)
Supplement: Table S1 — ERN source localization based on combined EEG/MEG data. ERN source localization based on combined EEG/MEG data. Maxima and locations of clusters where dipole sources were significantly different from zero. Clusterwise probabilities (CWP) are based on correction for the entire cortical surface. P-values are provided for the most significant dipole source in each cluster. Current direction in all clusters outwards from the cortical surface. (DOCX) [file pone.0101784.s002.docx]

Table S1

| Cortical Region | Cluster Size (mm^2^) | Approximate Talairach Coordinates | | | Brodmann Area | Max. p-value (-base-10 log) | CWP |
| --- | --- | --- | --- | --- | --- | --- | --- |
|  |  | x | y | z |  |  |  |
| **Healthy Participants** | | | | | | | |
| Left posterior cingulate sulcus | 1006 | -6 | -18 | 37 | 31 | 9.65 | .02 |
| Right posterior cingulate sulcus | 1042 | 10 | -13 | 36 | 31 | 8.44 | .005 |
| **Schizophrenia** | | | | | | | |
| Left posterior cingulate sulcus | 869 | -13 | -20 | 35 | 31 | 5.13 | .03 |
| Right posterior cingulate sulcus | 935 | 18 | -31 | 38 | 31 | 4.99 | .008 |
| **OCD** | | | | | | | |
| Left posterior cingulate sulcus | 884 | -14 | -15 | 37 | 31 | 5.84 | .02 |
| Left posterior cingulate gyrus | 674 | 9 | -30 | 38 | 23 | 5.79 | .02 |
